# Enhanced Transdermal Delivery of Granisetron by Using Iontophoresis

**Published:** 2012

**Authors:** Prabhakar Panzade, Ashish Heda, Prashant Puranik, Mayur Patni, Vipul Mogal

**Affiliations:** *Department of Pharmaceutics, Government College of Pharmacy, Aurangabad, Maharashtra, India.*

**Keywords:** Granisetron, Iontophoresis, Penetration enhancer, Poloxamer, Transdermal

## Abstract

The purpose of the present study was to explore the passive and electrically assisted transdermal transport of Granisetron hydrochloride (GRA) in solution and gel formulation through iontophoresis and also the feasibility of delivering therapeutic amounts of drug for the treatment of chemotherapy-induced nausea and vomiting. In this study, iontophoretic permeation of GRA through guinea pig skin using silver-silver chloride electrode was performed and the effects of different variables on this phenomenon were evaluated. Preliminary *in-vitro* studies using aqueous GRA formulations investigating the effect of drug concentration (5, 10, 15 and 20 mg mL^-1^) on passive permeation, current density (0.2, 0.4 and 0.5 mA cm^-2^), mode of current application, penetration enhancers and effect of application duration were performed. As expected, GRA delivery was found to be increased with the elevation in drug concentration and current density. Anodal continuous current delivery was more effective in the permeation of GRA than the pulsed current method. Penetration enhancers were ineffective to show synergistic effect in conjunction with iontophoresis. It was evident that reservoir in the skin was not formed during the iontophoretic delivery. The results confirm that GRA is an excellent candidate for iontophoresis. The present study demonstrated the feasibility of GRA transdermal transport through the Lutrol F-127 gel by iontophoresis. Further *in-vivo* studies will be required to support *in-vitro* conclusions and develop *in-vitro, in-vivo* correlations.

## Introduction

Prevention and control of nausea and vomiting is vital for the effective treatment of chemotherapy side effects in cancer patients. The administration of 5-hydroxytryptamine receptor antagonist has transformed the therapeutic approach to the acute and delayed emesis. However, effective control depends not only on the dose, but also on the duration of treatment and the route of administration. Both the oral and intravenous (IV) routes, which are routinely used, present disadvantages: IV administration requires qualified medical personnel and it is not practical for chronic treatment on an outpatient basis (for example, in the treatment of delayed emesis) whereas the oral route can be associated with erratic drug absorption, especially in patients suffering from nausea and prone to vomiting. Transdermal iontophoresis provides a non-invasive alternative for controlled drug delivery; the application of a small electric potential enables the transport of polar and charged drug molecules into the skin. In addition to expanding the range of drugs that can be delivered by this route, iontophoresis provides a tight control over the drug input kinetics and reduces intra- and inter-individual variability.

Granisetron (MW 348.9, K_o/w _0.38, pKa 9.4) is a selective 5-hydroxytryptamine-3 (5HT3) receptor antagonist shown to be effective in treating nausea and vomiting induced by cancer chemotherapy (1). Intravenous infusion of 40 µg/Kg granisetron is effective to prevent nausea and vomiting for repetitive chemotherapeutic regimens. Orally administered granisetron is also subjected to hepatic first pass effect and may be associated with certain unwanted gastrointestinal effects. Thus, there is a rationale for investigating the alternate delivery systems for enhanced efficacy, safety and increased patient compliance. Transdermal delivery can bypass the first pass metabolism and deliver the drugs in rate-controlled manner, which is desirable. Granisetron is very potent, hydrophilic in nature and has the first pass hepatic metabolism (2) that makes it a good candidate for transdermal iontophoretic delivery.

Iontophoresis involves the application of small amounts of current to push charged drug molecules through the skin (3, 4), resulting in higher fluxes of drug molecules for which permeation through skin is negligible otherwise. It provides the advantage of improved patient compliance, avoidance of first pass metabolism, controlled rate of drug release from the patch and the possibility of programmed delivery (5, 6). Some important considerations include: 1) flux proportionality should be applied to current density, 2) ions other than drug will decrease the efficiency of iontophoretic transport of the drug and 3) current up to 0.5 mA/cm^2^ is believed to be suitable for patient tolerance. Since the drug delivery is proportional to the applied current, a significant advantage of iontophoresis includes the possibility of pre-programming the delivery. The drug delivery may be dose-tailored on an individual basis or time-tailored in a constant or pulsatile fashion. The magnitude of a compound’s potential for iontophoretic delivery is theoretically proportional to its charge (7, 8) and inversely related to its molecular weight (9) and Hydrophobicity (10, 11). The onset of action with iontophoretic treatment is short, typically of the order of minutes and in contrast to hours by passive transdermal delivery.

Due to the complex nature of drug delivery, most of the studies related to the transdermal iontophoresis are focused on aqueous solutions (12). Gels are considered to be the most suitable delivery vehicles for iontophoresis, as they can be easily amalgamated with the iontophoretic delivery system and match the contour of the skin. Gels also have other advantages over liquids, such as ease of fabrication into the device, suitability with the electrode design, deformability into skin contours, better occlusion, and better stability. Moreover, the high proportion of water employed in gel formulations can in-turn provide an advantageous electroconductive base for clinical use (13). Lutrol is a polyoxypropylene-polyoxyethylene, non-ionic, surface-active block copolymer composed of ~70% ethylene oxide and 30% propylene oxide with an average molecular weight of 115 000 Da (14). The fact that poloxamer solution (20%-30% w/v in water) forms a reversible gel above 4^0^C (*i.e*. solution at low temperature and gel at higher temperature), offers a unique advantage of ease in handling and application. The reversible sol-gel property allows the cool solution to flow onto the skin and spread across it during its transformation to a nonocclusive gel at body temperature (15). Furthermore, owing to the poloxamer solution’s ability to form a hydrogel, it can show good electrical conductivity. In addition, this property can be exploited for the refillable unit dose iontophoretic drug delivery systems.

The present study was undertaken to assess the feasibility of delivering GRA using Lutrol F-127 as a vehicle for the iontophoretic transdermal delivery. The approach involved checking the drug permeability by passive and iontophoretic transport using an *ex-vivo* hairless guinea pig skin model. The effect of drug concentration, current density, pulsed current and various penetration enhancers were investigated.

## Experimental

Granisetron Hydrochloride and Lutrol F-127 were gift samples from Wockhardt, Aurangabad, India. Potassium dihydrogen phosphate and sodium hydroxide were purchased from Merck (Merck, India). Silver chloride was purchased from Research fine lab (Research fine lab, Mumbai). Silver wire (1 mm diameter, 99.9% pure) was purchased from local supplier. Other chemicals used in the study were of analytical grades and were purchased from Loba Chime, Research fine lab, Mumbai.

*Preparation of electrodes*

Silver-silver chloride electrodes were used for their stability and reversibility (16). The rod-shaped cathode was prepared through dipping the silver wire into the molten silver chloride to form thin and uniform coat. The electrodes were chlorinated by immersing in 0.1 M HCl (11).

*Skin preparation*

The density of the hair on human skin and guinea pig skin is similar (18). Hence, guinea pig skin was chosen for the permeation studies. Guinea pig which had been given free access to food and water was sacrificed using respiratory paralysis through chloroform, immediately before the experiment. The hair of the guinea pig skin at dorsal side was removed with hair remover clipper 24 h before the experiment. The skin was carefully excised; adhering fat and other visceral debris was removed manually. Separated skin was washed with saline solution before starting the experiment (19).

*Ex-vivo permeation study*

The modified glass diffusion cells (K-C cell) (Amol Scientific) were used for permeation studies. Freshly excised full thickness guinea pig skin was mounted between the donor and receptor compartments and securely clamped together. Skin was mounted so that stratum corneum could face towards the donor compartment. Available skin surface area for permeation was approximately 3.88 cm^2^. Excessive skin at the sides was trimmed off to minimize lateral diffusion. The receptor compartment was filled with 12 mL of phosphate buffer with pH of 7.4. Skin was allowed to equilibrate with the receptor fluid for 15 min.

The receptor solution was stirred by a star-head magnetic bar (size 10 × 10 mm) (Himedia) rotating at a constant speed of 600 rpm through a motorless magnetic mega stirrer (WHIRLMATIC-MEGA, Spectralab). The temperature in the bulk of the solution was maintained at a constant level of 37 ± 1°C using constant temperature water circulating bath (Deep Engineering), which circulates thermostated water through the water jacket surrounding the receptor compartment.

The donor phase was consisting of 5 mg/mL, 10 mg/mL, 15 mg/mL and 20 mg/mL of GRA to determine the effect of drug concentration on passive permeation. At the predetermined sampling intervals, aliquots of 1 mL were withdrawn periodically and replaced with the same volume of fresh receptor fluid. Each experiment was performed in triplicate. The withdrawn samples were suitably diluted and assayed spectrophotometrically at 302.80 nm. Further experiments were carried out to optimize the current density, pulsatile current and effect of various penetration enhancers.

*Preparation of thermoreversible gel*

Gels were prepared by the cold method (18). Gels containing 18%, 20%, and 22% w/v of Lutrol-F127 were prepared in distilled water to optimize the viscosity. Gel containing GRA and Lutrol-F127 was prepared as follows:

Exactly 100 mg of drug was dissolved in distilled water. It was constantly stirred using a Teflon-coated magnetic bead. Exactly 18%, 20% and 22% w/v of Lutrol-F127 were dispersed slowly into this drug solution and the resulting mixture was then refrigerated at 4°C for 24 h to obtain a completely hydrated, homogeneous and clear sol. Sol was removed from refrigerator and placed at room temperature, until it forms a completely hydrated, homogeneous and clear gel.

*Ex-vivo permeation study using thermoreversible gel*

Freshly excised full thickness guinea pig skin was mounted between the donor and receptor compartments and securely clamped together. Skin was mounted so as to face stratum corneum towards the donor compartment. The receptor compartment was filled with phosphate buffer (pH = 7.4). Skin was allowed to equilibrate with receptor fluid for 15 min. The temperature in the bulk of the solution was maintained at a constant level of 37 ± 1^0^C. Direct current of 0.5 mA/cm^2^ was applied for the period of 8 h. Samples (1 mL) were withdrawn from the receptor compartment at hourly interval for a period of 8 h and assayed for drug content by UV at 302.80 nm. Fresh phosphate buffer was added to replace the withdrawn sample volume. Passive permeation was checked without the application of any current.

**Table 1:** Permeation parameters for passive diffusion

**Drug concentration (mg/mL)**	**Q**_8_ **(mg/cm** ^2^ **)**	**Jss** **(mg/cm** ^2^ **/h)**	**Kp**
5	0.0962	0.00675 ± 0.0047	0.0004
10	0.1899	0.0149 ± 0.0039	0.0004
15	0.1931	0.0150 ± 0.00444	0.0005
20	0.2007	0.0157 ± 0.0035	0.0006

Jss = Steady state flux, Kp = Permeability coefficient, Er = Enhancement ratio, Q_8_ = Cumulative amount permeated in 8 h

Data Mean ± S.D.

*Sample collection and data analysis*

Exactly 1 mL of the sample was collected after every 1 h from the side arm of the diffusion cell using a syringe and was replaced with the same volume of pre-warmed (37^0^C) fresh receptor medium. The collected samples were sufficiently diluted and tested for the drug content at 302.80 nm using a UV spectrophotometer (Shimadzu 1700). The real steady-state situation was not observed clearly during the permeation studies. For this reason, the flux (J_ss_) was calculated from the slope of the linear portion of the curve. Q_8 _is the cumulative amount of drug permeated per cm^2^ of skin in 8 h. The enhancement ratio (Er) for the flux was calculated by using the following Equation 1:

Er = Iontophoretic Flux / Passive Flux (Equation 1)

Permeability coefficient (Kp) was calculated using the following Equation 2:

Kp = Flux / Initial amount of drug in donor compartment (Equation 2)

Statistical analysis of the data was analyzed by one-way analysis of variance (ANOVA) followed by Tukey-Kramer test, with the significance level set at 0.05. The data was expressed as mean ± SD (Graph pad INSTAT 3.01).

## Results and Discussion

*Ex-vivo passive permeation study of GRA to study the effect of drug concentration*

**Figure 1:**
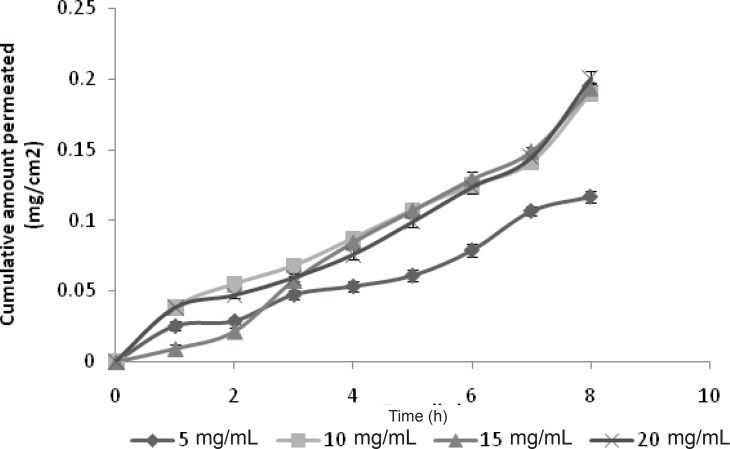
Effect of drug concentration

The aim of this study was to evaluate the effect of drug concentration on the permeation profile. The study was carried out at 5 mg/mL, 10 mg/mL, 15 mg/mL and 20 mg/mL of drug concentration. An increase in the flux with increasing donor concentration was observed, however, but reached plateau after 10 mg/mL donor concentration (Figure 1). However, no significant difference in flux was observed for higher drug concentration (p > 0.05) (Table 1). The cumulative amounts delivered for 5 mg/mL and 10 mg/mL donors were significantly different (p < 0.05) but not for 10 mg/mL and 20 mg/mL donors.

This fact may be attributed to the saturation of skin at higher concentration of drug which leads to the less permeation. A non-linear relationship between the flux and the concentration of nicotine has been reported for this reason (20). Hence, the drug concentration of 10 mg/mL was selected as the optimized drug concentration for the further iontophoretic study.

*Ex-vivo permeation study of GRA to optimize the current density*

**Figure 2:**
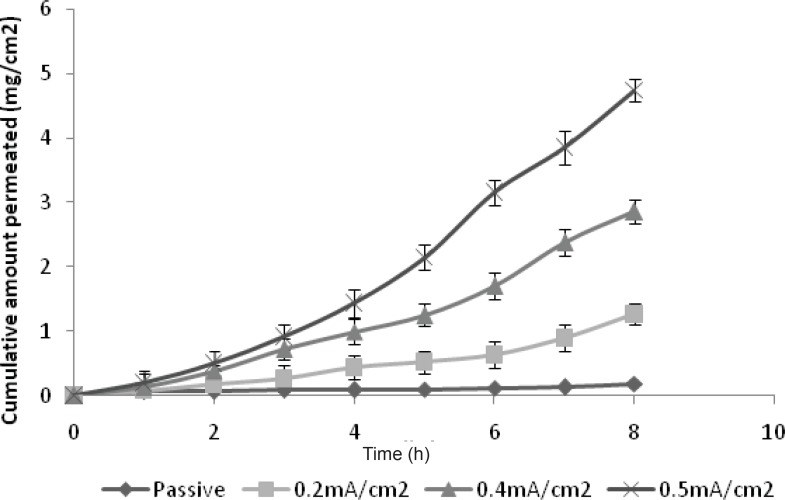
Optimization of current density

The optimization of current density is essential for the maximum permeation of drug since the permeation of drug is directly affected by the quantity of current applied. Hence, the permeation studies were carried out at three different current densities of 0.2, 0.4 and 0.5 mA/cm^2^. It was observed that an increase in current density results in the increased permeation of GRA as indicated in Figure 2. It can be discerned that the relationship between flux and current density is linear. A direct proportionality between the current density and the ion flux is a general characteristic of iontophoresis, predicted by the Nernt-Planck equation (5). The current density of 0.5 mA/cm^2^ was reported to be the maximum tolerable current density by human being. As the current density was progressively increased, flux was also increased (Table 2). The cumulative amount permeated for 0.5 mA/cm^2^ current density was significantly high compared to 0.2 mA/cm^2^ (p < 0.05). At 0.2 mA/cm^2^ current density, the cumulative amount permeated was 1.2654 mg/cm^2^ while at 0.5 mA/cm^2^, which was 4.7459 mg/cm^2^, showed 3.8 fold increases in permeation. This may be attributed to the fact that an increase in current density may cause an increase in the pore transport of the drug. This involves the opening of more sweat duct resulting in more number of pores at higher current density as the pore pathway is one of the pathways assumed for iontophoresis. Since the maximum permeation was observed at 0.5 mA/cm^2^, the same current density was used for further study.

There was also significant increase in flux with Iontophoretic permeation as compared with the Passive permeation (p < 0.05) since the drug has a low permeability due to its hydrophilic nature, which results in less passive permeation. The flux of iontophoretic permeation study (0.5 mA/cm^2^) was 0.6322 mg/cm^2^/h, while it was only 0.0149 mg/cm^2^/h for the passive study, which showed 42.5 fold increases in flux with iontophoresis.

*Ex-vivo permeation study of GRA to study the effect of pulsatile current*

The use of continuous direct current may result in permanent skin polarization, which can reduce the efficiency of iontophoretic delivery proportional to the length of direct current application. The buildup of this polarizable current can be overcome by using pulsed direct current that is delivered periodically.

**Figure 3:**
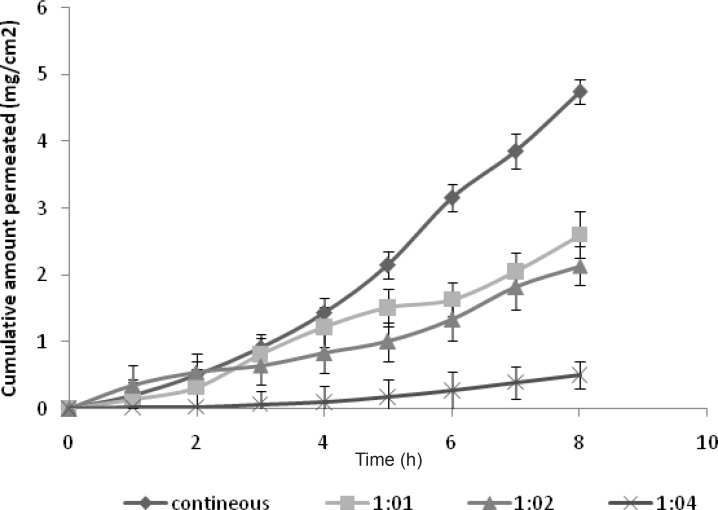
Effect of pulsatile current

The permeation of drug was studied in the pulse ratio of 1:1, 1:2 and 1:4. The utmost permeation was found for the pulse ratio of 1:1 in comparison with the higher pulse ratio (Figure 3). This may be due to the fact that at higher pulse ratio, the skin remains in a polarized condition more than the other times. However, in the present study, it was observed that the direct current was more efficient than pulse current to promote GRA permeation (p < 0.05). The flux of GRA decreased with pulse current 1:1 (flux = 0.4189 ± 0.0934 mg/cm^2^/h) when compared with the continuous direct current of the same current intensity (flux = 0.6322 ± 0.0199 mg/cm^2^/h) (Table 2).

**Table 2:** Effect of various variables on permeation parameters

**Variables**		**Q**_8 _ **(mg/cm** ^2^ **)**	**Jss (mg/cm** ^2^ **/h)**	**Kp**	**ER**
**Current Density (mA/cm** ^2^ **)**	**Passive**	0.1899	0.0149 ± 0.0039	0.0004	1.0
	**0.2**	1.2654	0.1170 ± 0.167	0.0039	7.8523
	**0.4**	2.8587	0.3169 ± 0.177	0.0105	21.2684
	**0.5**	4.7459	0.6322 ± 0.207	0.0218	42.4295
**Pulse Ratio** **ON:OFF**	**1:1 (1sec)**	2.6070	0.4189 ± 0.287	0.0161	28.1140
	**1:2 (1sec)**	0.2140	0.4120 ± 0.300	0.0137	27.6510
	**1:4 (1sec)**	0.5090	0.0983 ± 0.229	0.0032	6.5973
**Penetration Enhancer**	**DMSO (5%)**	3.7882	0.5630 ± 0.0224	0.0187	37.5872
	**Ethanol (5%)**	4.0873	0.5728 ± 0.0652	0.0226	45.6375
	**Tween-80 (5%)**	3.5818	0.5435 ± 0.0384	0.0197	39.8255
	**PEG-400 (5%)**	4.4079	0.6213 ± 0.0863	0.0243	49.0805
**Current Application (Hr)**	**0**	0.1899	0.0149 ± 0.0039	0.0004	3.0755
	**2**	0.6587	0.1624 ± 0.205	0.0108	10.8993
	**4**	1.0436	0.1860 ± 0.281	0.0124	12.4832
	**8**	4.7459	0.6322 ± 0.249	0.0218	42.4295
**Formulation**	**Passive Solution**	0.1899	0.0149 ± 0.207	0.0005	1.0
	**Passive gel**	0.0755	0.0075 ± 0.005	0.0004	1.0
	**Ionto solution**	4.7459	0.6322 ± 0.0039	0.0218	42.4295
	**Ionto gel**	2.2266	0.2917 ± 0.148	0.0097	19.5771

The observed increased efficiency of the direct current over the pulse current can be explained through the fact that in latter case, the quantity of electric charge permeation through the skin is reduced by half as a function of square wave current. In addition, the pulsed current was considered to be less damaging to the skin. During the application of continuous direct current and subsequent polarization, the smaller molecules like GRA may escape through the paths of low skin impedance (like skin appendages: hair follicles and sweat glands).

*Ex-vivo permeation study of GRA to study synergistic effect of penetration enhancers*

Passive permeation of drugs across the skin can be increased with transdermal penetration enhancers, as the tightly organized bilayer structure of the skin is weakened. One way to increase the drug penetration is to add the enhancer to the drug formulation. Another way, is to pre-treat the skin with the enhancer before the drug application.

**Figure 4:**
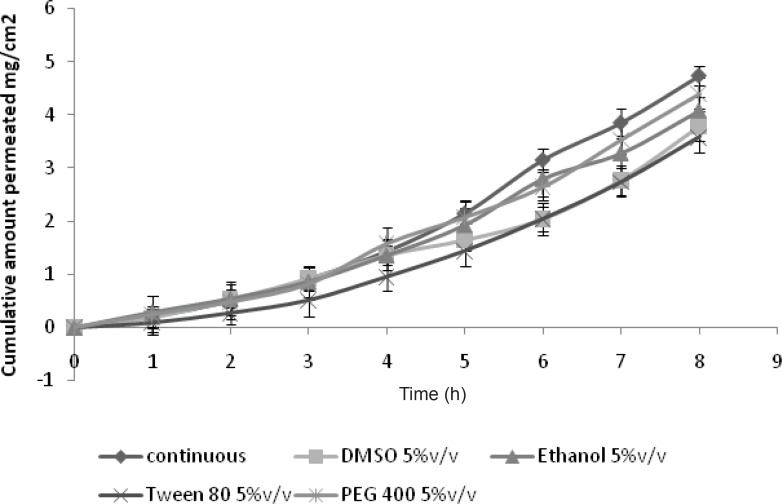
Effect of penetration enhancer

It was found that no penetration enhancer was able to increase the permeation over the iontophoresis alone (Figure 4); this might be due to the fact that the maximum current density has been used with the iontophoresis. Penetration enhancers were ineffective to give the synergistic effect with the iontophoresis as these two modalities may act by using the different pathway or mechanism. Only PEG-400 has given the comparable results with that of continuous current (p > 0.05) (Table 2).

*Ex-vivo permeation of GRA to study the effect of duration of current application*

**Figure 5:**
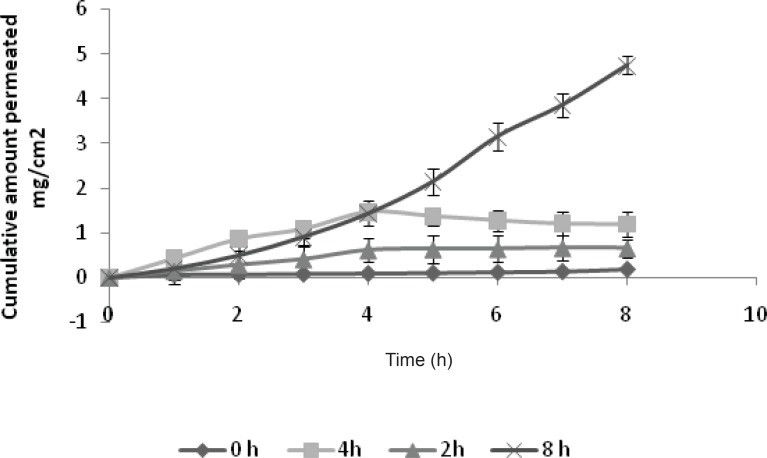
Effect of duration of application of current

Figure 5 shows the effect of the application time of the electrical field on the cumulative amount of drug permeated. The results indicate that the permeation profile of drug increases with an increase in the duration of application of current. Without applying the current, the rate of drug permeation was low. Flux was greater when the iontophoresis was applied for 8 h instead of 2 h. Termination of current did not cause the flux to return immediately to the passive control level in both 2 h and 4 h treatment. The data obtained is shown in Table 2.

When the current was applied for 2 h and 4 h and then terminated, the cumulative quantity of GRA detected in receptor compartment did not increase linearly with time; but the flux remained almost constant over a period of time. This indicates that there is no possibility of formation of reservoir in the skin during the iontophoresis and the drug continues to flow at constant rate. The results are in a good agreement with researchers working with ketorolac (21).

*Preparation and evaluation of gel formulation containing GRA*

Lutrol-F127 was selected since it forms a gel with acceptable viscosity, clarity and release characteristics. Lutrol-F127 usually forms a thermoreversible gel. A mass of 1% w/v GRA gel with 18% w/v, 20% w/v and 22% w/v of Lutrol-F127 were prepared by standard procedure and further evaluated for the following parameters.

The viscosity of the polymeric dispersion containing 18%, 20%, and 22% w/v of Lutrol-F127 in distilled water was reported to assess their gelling characteristics. It demonstrates that an increase in the concentration of Lutrol-F127 increases the gelling property of the polymer. The polymeric dispersions containing 22% w/v of Lutrol-F127 had the highest viscosity (9986 ± 53 cp) at the room temperature. The dispersion containing 18% w/v of Lutrol-F127 formed a gel with very low viscosity (1440 ± 21cp) which indicates the less concentration of gelling agent. At 20% w/v of Lutrol-F127, the gel was formed with a good viscosity (9224 ± 45cp) and clarity. Thus, 20% w/v of Lutrol-F127 was selected as an optimum concentration of gelling agent for further studies. It had been observed that the gel so prepared with 20% w/v Lutrol-F127, had sufficient viscosity to hold the formulation in the electrode cavity when the electrode was applied to the skin. These gels were evaluated for the viscosity, clarity and pH and the evaluation parameters are as shown in Table 3.

**Table 3:** Evaluation of Lutrol- F127 gels

**Formulation code**	**pH**	**Viscosity (cp)**	**Clarity**
A	5.77 ± 0.11	1440 ± 21	++
B	6.98 ± 0.12	9224 ± 45	+++
C	6.63 ± 0.16	9986 ± 53	++

*Ex-vivo permeation study using GRA gel formulation*

**Figure 6:**
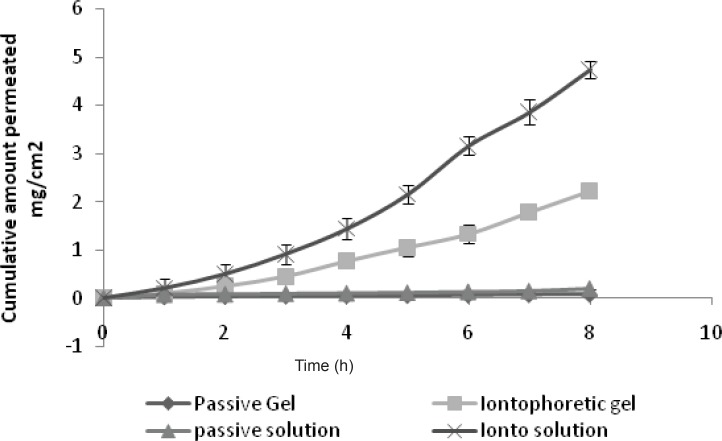
Comparison of solution and gel

The study reveals that significant difference was observed with the iontophoretic permeation to passive the permeation. The flux of iontophoretic study was found to be 0.2917 mg/cm^2^/h, and it was only 0.0075 mg/cm^2^/h for the passive permeation (Table 2). The change in viscosity greatly modified the iontophoretic transport of GRA. The flux significantly decreased for both the iontophoretic and passive study using gel formulation compared with the aqueous solution (p < 0.05) (Figure 6). The reason for this may be the viscosity of gel. Increased viscosity reduced the mobility of ions and also prevented the homogenization of donor phase, requiring a higher driving force for the ions to move (22).

## Conclusions

The results confirm that GRA is an excellent candidate for iontophoresis. It has also been shown that its transdermal flux basically increases linearly as a function of GRA concentration by passive permeation study. Lutrol F-127 could be used to formulate a thermosensitive gel for iontophoresis that will gel upon application to skin. However, continuous current produced more permeation and flux than the pulsatile iontophoresis. The results showed that penetration enhancers were not effective in conjunction with iontophoresis. The present study demonstrated the feasibility of GRA transdermal transport through Lutrol F-127 gel by iontophoresis. Further *in-vivo* studies will be required to support *in-vitro *conclusions and develop *in-vitro, in-vivo *correlations.
